# Single-cell analyses of X Chromosome inactivation dynamics and pluripotency during differentiation

**DOI:** 10.1101/gr.201954.115

**Published:** 2016-10

**Authors:** Geng Chen, John Paul Schell, Julio Aguila Benitez, Sophie Petropoulos, Marlene Yilmaz, Björn Reinius, Zhanna Alekseenko, Leming Shi, Eva Hedlund, Fredrik Lanner, Rickard Sandberg, Qiaolin Deng

**Affiliations:** 1 Department of Cell and Molecular Biology, Karolinska Institutet, 171 77 Stockholm, Sweden;; 2 School of Pharmacy, Fudan University, 201203 Shanghai, China;; 3 Department of Clinical Science, Intervention and Technology and Division of Obstetrics and Gynecology, Karolinska Universitetssjukhuset, 14186 Stockholm, Sweden;; 4 Department of Neuroscience, Karolinska Institutet, 171 77 Stockholm, Sweden;; 5 Ludwig Institute for Cancer Research, 171 77 Stockholm, Sweden

## Abstract

Pluripotency, differentiation, and X Chromosome inactivation (XCI) are key aspects of embryonic development. However, the underlying relationship and mechanisms among these processes remain unclear. Here, we systematically dissected these features along developmental progression using mouse embryonic stem cells (mESCs) and single-cell RNA sequencing with allelic resolution. We found that mESCs grown in a ground state 2i condition displayed transcriptomic profiles diffused from preimplantation mouse embryonic cells, whereas EpiStem cells closely resembled the post-implantation epiblast. Sex-related gene expression varied greatly across distinct developmental states. We also identified novel markers that were highly enriched in each developmental state. Moreover, we revealed that several novel pathways, including PluriNetWork and Focal Adhesion, were responsible for the delayed progression of female EpiStem cells. Importantly, we “digitalized” XCI progression using allelic expression of active and inactive X Chromosomes and surprisingly found that XCI states exhibited profound variability in each developmental state, including the 2i condition. XCI progression was not tightly synchronized with loss of pluripotency and increase of differentiation at the single-cell level, although these processes were globally correlated. In addition, highly expressed genes, including core pluripotency factors, were in general biallelically expressed. Taken together, our study sheds light on the dynamics of XCI progression and the asynchronicity between pluripotency, differentiation, and XCI.

ESCs are an important cellular resource for studying mammalian embryonic development. mESCs maintained either in a conventional serum/LIF condition or in a ground state 2i condition are considered to exhibit a naïve state of pluripotency (Bradley et al. 1984; Nichols and Smith 2009, 2011). Ground state mESCs have a more homogenous transcriptional and morphological profile and exhibit higher expression of pluripotency genes, including *Nanog* and *Prdm14*, than conventional mESCs (Chambers et al. 2007; Yamaji et al. 2013). Initial experiments on *Nanog* suggested that the control of pluripotency is determined by biallelic expression in the ground state 2i condition versus monoallelic expression in the conventional serum/LIF condition (Miyanari and Torres-Padilla 2012). However, this was soon questioned by two subsequent studies that observed consistent biallelic *Nanog* expression in mESCs (Faddah et al. 2013; Filipczyk et al. 2013). The allelic expression pattern of pluripotency factors remains unresolved, as does its possible role in regulating stem cell states. In contrast to mESCs, mouse EpiStem cells (mEpiSCs) represent a primed developmental state of pluripotency, defined by their propensity for differentiation and random XCI, representing a suitable model for post-implantation development (Brons et al. 2007; Tesar et al. 2007).

Random XCI is a crucial event during the development of female mammals (Schulz and Heard 2013). Random XCI occurs shortly after implantation, and differentiating ESCs are regarded as a useful tool to study XCI, as they recapitulate multiple events occurring during early development (Heard 2004; Pollex and Heard 2012). XCI can be associated with the differentiated cell state via interaction of pluripotency genes with two major long noncoding RNAs *Xist* and *Tsix* (Navarro et al. 2008; Nesterova et al. 2011). It is generally accepted that both X Chromosomes remain active in mESCs grown in the ground state 2i culture condition, whereas random XCI occurs to varying degrees in mESCs grown in the conventional serum/LIF condition (Schulz et al. 2014). However, single-cell allelic gene expression analyses that correlate pluripotency, differentiation, and XCI are currently lacking.

Here, we systematically characterized the transcriptomic profiles of male and female mESCs across different developmental states using single-cell RNA sequencing (RNA-seq) with allelic resolution. Specifically, we investigated the relationship between pluripotency, differentiation, and XCI dynamics, and the genes and pathways associated with the delayed progression of female EpiSCs. We also examined allelic gene expression including pluripotency genes and found that the allelic patterns of genes generally reflect their expression levels.

## Results

### mESCs display distinct transcriptional profiles along developmental progression

**Figure 1.**
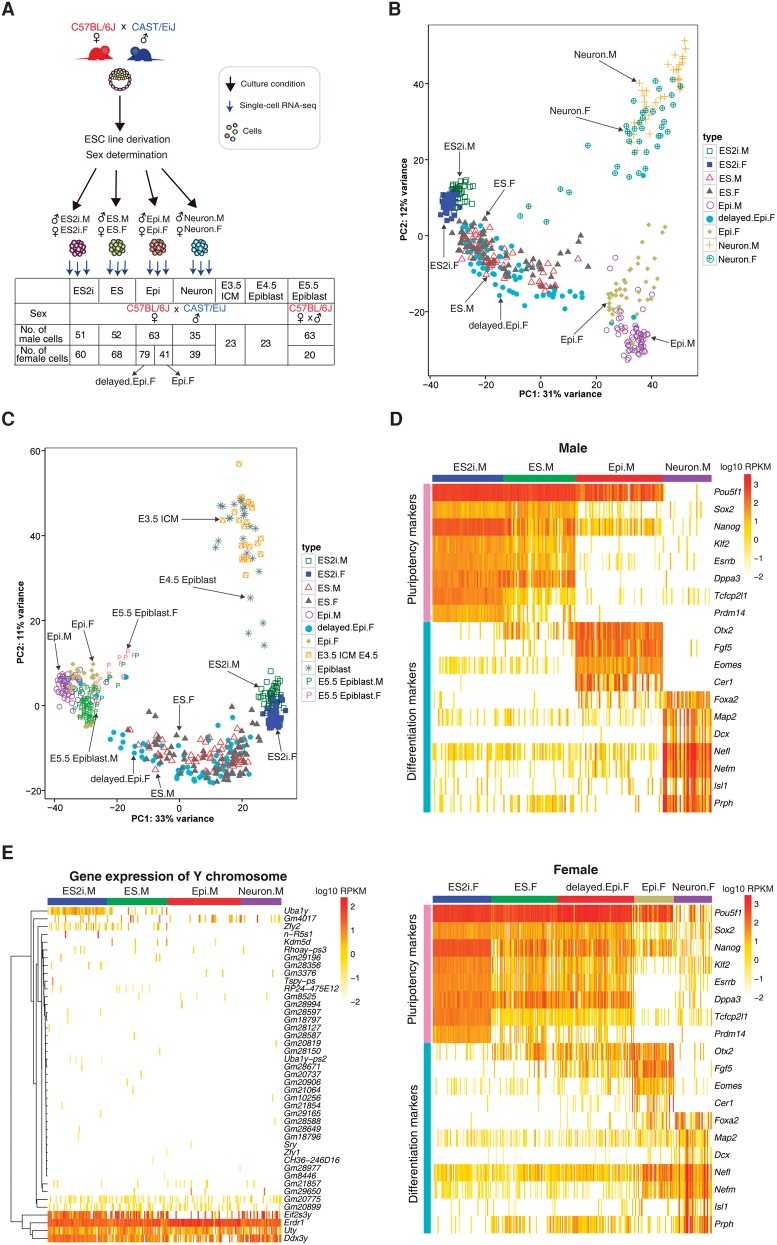
Gene expression profile of in vitro and in vivo mouse embryonic cells. (*A*) Experimental design of this study: (ICM) inner cell mass. (*B*) PCA of the cultured embryonic stem cells based on the top 500 variable genes. (*C*) PCA of cultured, preimplantation (E3.5 ICM and E4.5 epiblast) and post-implantation (E5.5 epiblast) embryonic cells based on the top 500 variable genes. (*D*) Expression profile of pluripotency and differentiation genes in different conditions for male and female cells (*upper* and *lower* panels, respectively). (*E*) Y Chromosome gene expression across all male cells. (M) male; (F) female.

To study the developmental progression of mESCs with allelic resolution, we generated male and female mESCs derived from outbred E4 blastocysts (female C57BL/6J × male CAST/EiJ) (Fig. 1A). mESCs were cultured in 2i and LIF as the ground state condition or in serum and LIF as the conventional condition. mEpiSCs and post-mitotic neurons were also generated to study more advanced development. Hereafter, we designated these four conditions as ES2i, ES, Epi, and Neuron, respectively. We also obtained E3.5 inner cell mass (ICM), E4.5 epiblast cells, and post-implantation E5.5 epiblast cells (Fig. 1A). We sequenced the resulting single cells using the Smart-seq2 protocol (Picelli et al. 2013, 2014) and analyzed a total of 617 cells that passed the quality control (Methods).

Cells from each culture condition could be clearly distinguished by principal component analysis (PCA) based on the top 500 variable genes in expression reflecting their distinct developmental states (Fig. 1B). Similar clustering profiles were also observed using the top 200 and 1000 variable genes (Supplemental Fig. S1A,B). We generated two stages of Epi female cells that were designated as delayed.Epi.F and Epi.F, which differed only in culturing time (Methods). Interestingly, delayed.Epi.F cells clustered with ES, whereas Epi.F stayed close to Epi male (Epi.M) cells (Fig. 1B). Several female Neuron cells (Neuron.F) also exhibited slower differentiation compared to male Neuron cells (Neuron.M) with a few cells dispersed toward ES cells (further analyzed in a later section).

To relate in vitro ESCs to the corresponding in vivo embryonic stages at single-cell resolution, we compared the transcriptomes of preimplantation E3.5 ICM and E4.5 epiblast cells with our cultured mESCs. We found that ES2i cells were closer to the in vivo blastocyst cells in the PCA (Fig. 1C). Moreover, E4.5 epiblast cells had the trend of approaching ES2i, which suggested that mature epiblast cells were more similar to ES2i cells. Intriguingly, male and female post-implantation E5.5 epiblast cells clustered tightly together with Epi.M and Epi.F cells but not with delayed.Epi.F cells, further indicating the slower progression of delayed.Epi.F cells (Fig. 1C; see Supplemental Fig. S1C,D for PCA based on the top 200 and 1000 variable genes).

Moreover, pluripotency markers were highly expressed in both male (ES2i.M) and female (ES2i.F) cells, and their expression levels were gradually reduced with developmental progression. In line with the PCA plot, the gene expression profile for delayed.Epi.F was very similar to that of ES.F. Concomitantly, differentiation markers such as *Otx2* and *Fgf5*, showed higher expression levels in Epi.M cells and Epi.F cells, whereas post-mitotic neuron markers (e.g., *Map2*, *Nefl* and *Isl1*) were enriched in the Neuron condition (Fig. 1D).

We also found that except for a few ubiquitously expressed Y Chromosome genes such as *Erdr1*, *Uty*, and *Ddx3y*, *Sry* was not expressed even in the more differentiated Neuron state (Fig. 1E). Notably, *Uba1y* was expressed in ES2i.M cells, but rarely detected in other states of male cells (Fig. 1E).

### Enriched gene expression in each developmental state

**Figure 2.**
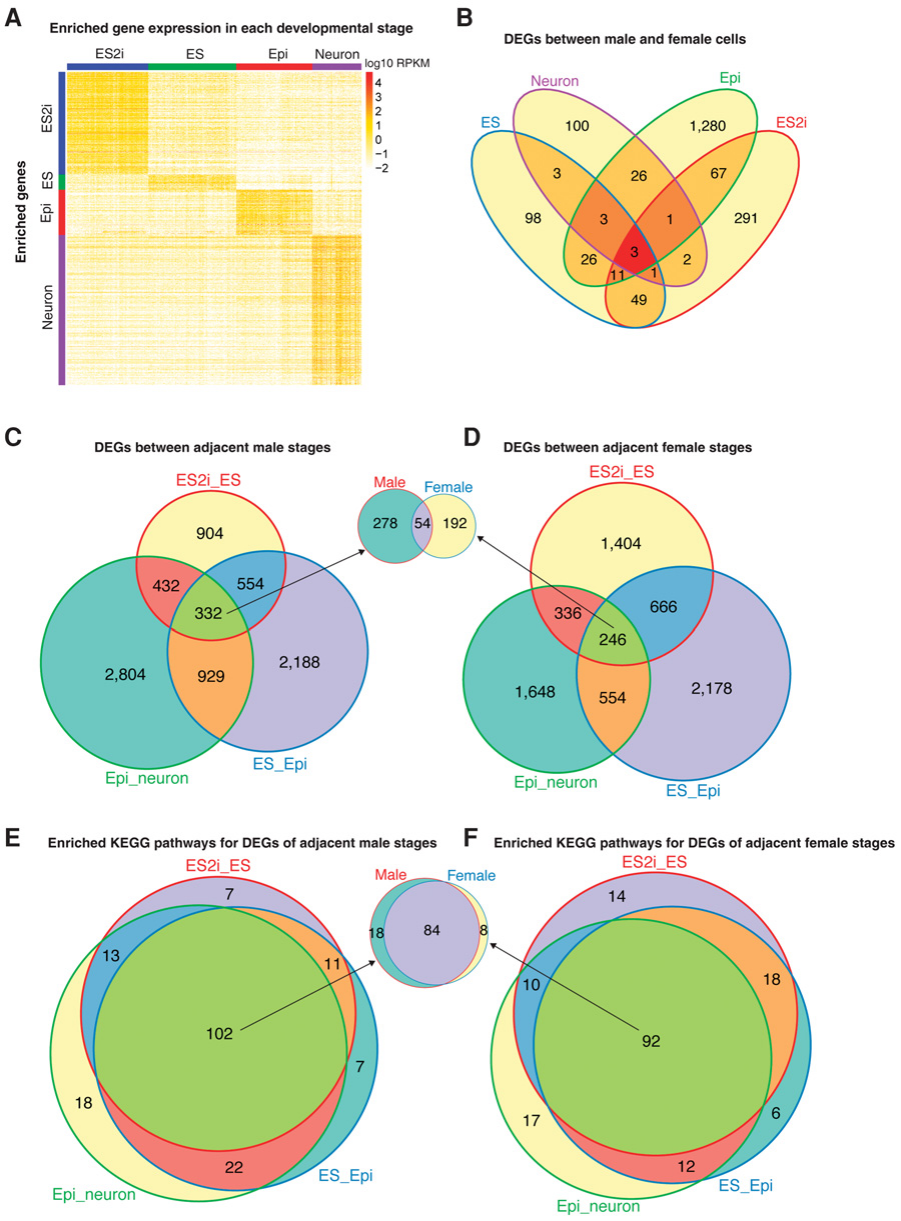
Differential gene expression analysis across developmental states. (*A*) Enriched gene expression profile in each developmental state. For the Epi condition, Epi.F cells but not delayed.Epi.F cells were used in order to match the developmental state. One-way ANOVA pairwise comparison was applied to the normalized gene expression of different conditions (including both male and female cells) to identify genes with enriched expression. Cutoff: fold change >4 and adjusted *P* < 0.01. (*B*) Differentially expressed genes between male and female cells among distinct conditions. (*C*,*D*) Differentially expressed genes between adjacent male/female states. (*E*,*F*) Enriched KEGG pathways for the DEGs of adjacent male/female states.

We applied one-way ANOVA pairwise comparison and separately identified 879, 130, 388, and 1286 genes that showed highly enriched expression in conditions of ES2i, ES, Epi, and Neuron (fold change >4 and adjusted *P* < 0.01) (Fig. 2A; Supplemental Tables S1–S4). Among these state-enriched genes, 86, 11, 38, and 77 were transcription factors (TFs) in each condition. Reassuringly, pluripotency markers such as *Nanog*, *Esrrb*, *Tcfcp2l1*, *Prdm14*, and *Klf2* were enriched in ES2i cells. Differentiation markers, including *Otx2*, *Fgf5*, *Eomes*, and *Cer1* were highly expressed in Epi cells, whereas post-mitotic neuron markers *Nefl*, *Nefm*, *Isl1*, and *Prph* were identified in the Neuron condition. Apart from these known markers, we also identified multiple novel markers. Pathway enrichment analysis suggested that state-specific enriched genes were mainly involved in metabolic pathways, MAPK signaling, Wnt signaling, and ErbB signaling pathways (adjusted *P* < 0.01) (Supplemental Fig. S2A–D).

### Sex-related differential gene expression across developmental states

To identify sex-related gene expression differences, we performed differential expression analysis between male and female cells of each condition using SCDE (Kharchenko et al. 2014). Here, Epi.F but not delayed.Epi.F cells were used to compare with Epi.M cells to match the developmental state. In total, 425 (30 TFs), 194 (11 TFs), 1417 (105 TFs), and 139 (9 TFs) differentially expressed genes (DEGs) were detected in ES2i, ES, Epi, and Neuron conditions, respectively (adjusted *P* < 0.01) (Fig. 2B). Only three DEGs overlapped across the four different conditions, of which all were Y Chromosome genes (*Uty*, *Ddx3y*, and *Eif2s3y*). These results suggest that sex-related gene expression varies greatly across distinct developmental states. Gene ontology (GO) enrichment analysis showed that these genes were mainly involved in developmental and metabolic biological processes (adjusted *P* < 0.01) (Supplemental Fig. S3A–D).

Moreover, 2222, 4003, and 4497 DEGs were detected in ES2i versus ES, ES versus Epi, and Epi versus Neuron, respectively (Fig. 2C), whereas 2652, 3644, and 2784 were identified in the female comparisons (Fig. 2D). Among these, 332 and 246 genes were common across all adjacent male and female comparisons, respectively. Fifty-four of these genes were shared between male and female cells (Fig. 2C,D). Although the majority of DEGs detected in adjacent male and female states were different, most of the significantly enriched KEGG pathways were the same (adjusted *P* < 0.01) (Fig. 2E,F). Moreover, 84 enriched KEGG pathways were shared across all male and female comparisons, including pathways of metabolism, MAPK signaling, Wnt signaling, and GnRH signaling (adjusted *P* < 0.01).

### XCI states vary greatly among female cells of each condition

**Figure 3.**
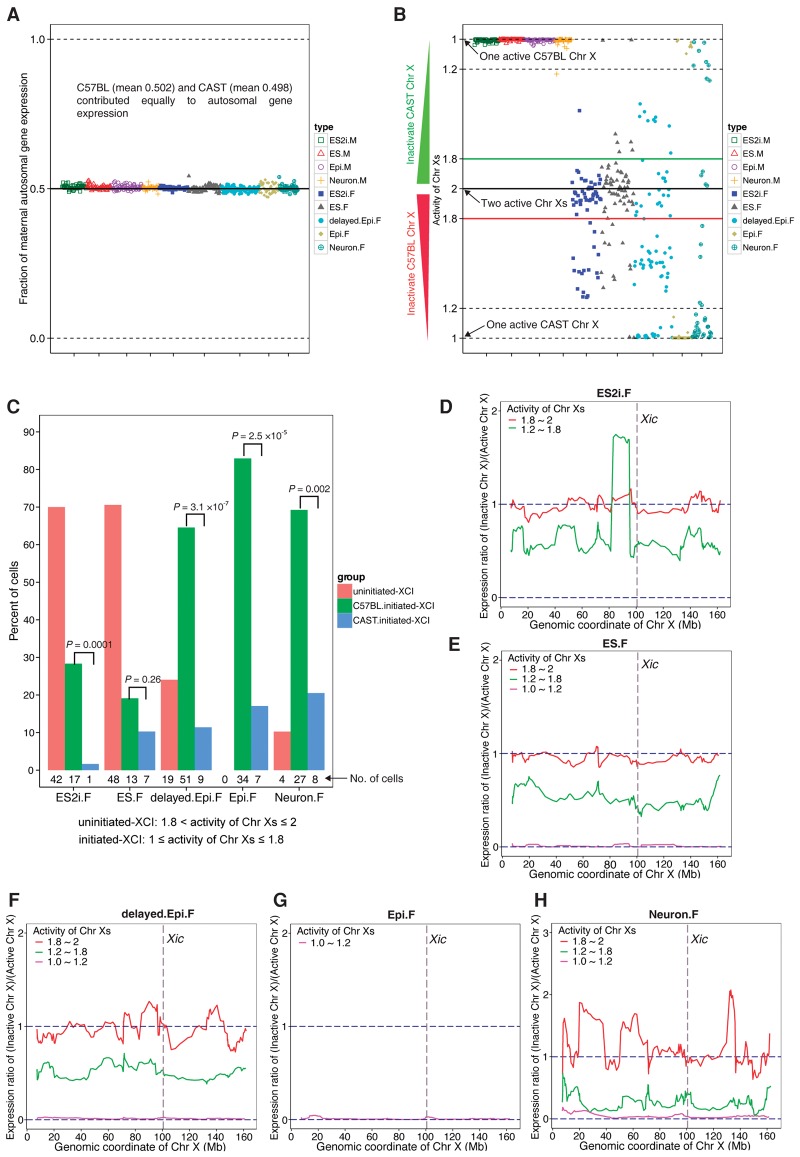
Dynamic and heterogeneous XCI states in female cells. (*A*) Fraction of maternal expression for autosomal genes in each condition of cells. Fraction of maternal expression was calculated using maternal allelic reads divided by the sum of maternal and paternal allelic reads. (*B*) Activity of maternal and paternal X Chromosomes in each condition. The indexes of the activity of Chr Xs in female cells range from 1 (one active X Chromosome) to 2 (two active X Chromosomes). Each type of female cell was divided into three different XCI groups: (1) uninitiated-XCI (1.8< activity of Chr Xs ≤2); (2) ongoing-XCI (1.2< activity of Chr Xs ≤1.8); and (3) completed-XCI (1≤ activity of Chr Xs ≤1.2). (*C*) The percentage of cells in each XCI group for each condition. Because it is not possible to determine whether the uninitiated-XCI cells are going to inactivate the maternal or paternal X Chromosome, Student's *t*-test was only applied to the cells of the initiated (ongoing and completed) XCI group. (*D*–*H*) Chromosome-wide expression ratio of inactive X Chromosome compared to active X Chromosome. The ratio was calculated using the number of allelic reads of inactive X Chromosome divided by that of active X Chromosome based on a moving window of an average of 10 genes. Red, green, and magenta lines denote the female cells in uninitiated-, ongoing-, and completed-XCI groups, respectively.

To explore XCI during developmental progression in individual female cells, we calculated the proportion of transcripts expressed from the maternal (C57BL/6J) and paternal (CAST/EiJ) X Chromosomes using our robust allelic calling pipeline (Deng et al. 2014) (Methods). First, we confirmed that the maternal and paternal allele contributed equally to gene expression of autosomes (mean 50.2% and 49.8%, respectively) (Fig. 3A). Moreover, expression of only the maternal X Chromosome was detected in all male cells with a mean of 99.2% reads mapped to the C57BL/6J allele (Fig. 3B). In order to “digitalize” the activity of the two X Chromosomes (activity of Chr Xs) in each cell, we converted the fraction of maternal X Chromosome expression to the range of 1 (XCI finished with one active X Chromosome) to 2 (XCI uninitiated with two active X Chromosomes) (Fig. 3B) (Methods). Furthermore, to better characterize XCI progression, we categorized female cells into three groups: (1) uninitiated-XCI (1.8< activity of Chr Xs ≤2); (2) ongoing-XCI (1.2< activity of Chr Xs ≤1.8); and (3) completed-XCI (1≤ activity of Chr Xs ≤1.2).

Surprisingly, we observed large heterogeneity in XCI progression within each developmental state (Fig. 3B). Most ES2i cells fell into the uninitiated-XCI group. However, a subpopulation of cells (∼30%) had already started to inactivate one X Chromosome, but none of these had completed XCI. In the ES condition, XCI states of female cells also varied tremendously, and 6% of ES.F cells had completed XCI (Fig. 3B,C). A significantly higher fraction of delayed.Epi.F cells displayed ongoing (50.6%) or completed (25.3%) XCI, whereas all Epi.F cells had completed XCI (Fig. 3B,C). As expected, the majority of Neuron.F cells showed completed (71.8%) or ongoing (17.9%), and only four cells displayed uninitiated XCI, reflecting the asynchronized differentiation (Fig. 3B,C). Intriguingly, we found that XCI preferentially occurred on the maternal X Chromosome of female cells in all culture conditions, leaving the paternal allele to be expressed (Fig. 3C). These data confirm the dominance of the CAST-derived X Chromosome (Chadwick et al. 2006). Collectively, these results demonstrate that female cells exhibit profound heterogeneity in XCI states even in the ES2i condition.

### X Chromosome wide progression of XCI

To further examine the X Chromosome-wide dynamics of XCI in female cells, we calculated the expression ratio of inactive X Chromosome to active X Chromosome using a moving window size of 10 genes on average (Methods) (Supplemental Table S5). As expected, gene expression from the inactive X Chromosome was comparable to that of the active X Chromosome in each condition in the uninitiated-XCI group (ratio ≈ 1) (red line in Fig. 3D,E,F,H). A larger variability was observed in Neuron.F cells, likely because only four Neuron.F cells were in the uninitiated-XCI group. Compared to the uninitiated-XCI group, the expression ratio of inactive X Chromosome in ongoing-XCI cells was reduced across the whole X Chromosome in all states, reflecting that XCI was taking place (green line in Fig. 3D,E,F,H). Notably, the *Apoo* gene nearby the X Chromosome inactivation center (*Xic*) on the inactive X Chromosome had a higher expression level than that of the active X Chromosome in the ongoing-XCI group of ES2i.F cells (Fig. 3D, the protruding region of green line) and *Apoo*’s ortholog has been reported as an escapee gene in humans (Zhang et al. 2013). As expected, the expression from the inactive X Chromosome was around 0 across the whole X Chromosome in the completed-XCI group (magenta line in Fig. 3E–H). Intriguingly, the expression levels of known frequent escapees such as *Kdm5c*, *Kdm6a*, *Ddx3xI*, and *2610029G23Rik* were gradually decreased with XCI progression, but there was still detectable expression in completed-XCI cells (Supplemental Fig. S4). Therefore, we conclude that XCI progressed along the entire X Chromosome.

### XCI is globally associated with pluripotency and differentiation but asynchronized at the single-cell level

**Figure 4.**
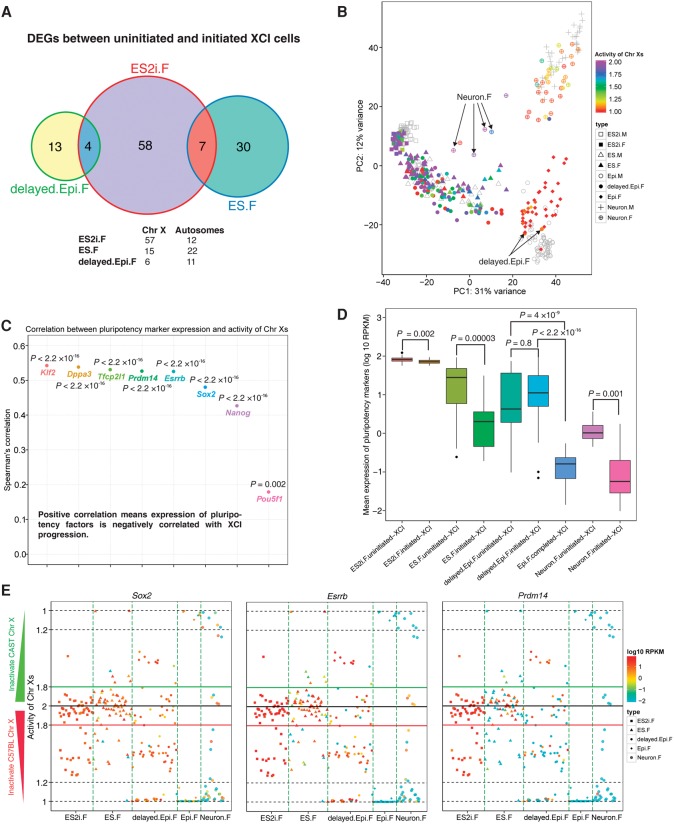
Association between pluripotency, differentiation, and XCI progression across different developmental states. (*A*) Venn graph for DEGs between uninitiated- and initiated-XCI cells. Female cells were divided into two distinct XCI state groups: (1) uninitiated-XCI (1.8< activity of Chr Xs ≤2); and (2) initiated-XCI (1≤ activity of Chr Xs ≤1.8). Adjusted *P* < 0.01. (*B*) Each female cell is colored by the corresponding activity of Chr Xs in PCA. Male cells are shown in gray. Two delayed.Epi.F cells with completed-XCI (arrows, 1≤ activity of Chr Xs ≤1.2) clustered together with Epi.M cells. Four Neuron.F cells with uninitiated-XCI (arrows) were closer to ES cells but further away from other Neuron.F cells. (*C*) Spearman's correlation between expression of pluripotency genes and the activity of Chr Xs. (*D*) Comparison of pluripotency gene expression between cells of uninitiated- and initiated-XCI groups for each condition. Epi.F only contained cells from completed-XCI group (1≤ activity of Chr Xs ≤1.2). The *y*-axis represents mean expression of eight pluripotency genes (*Pou5f1*, *Sox2*, *Nanog*, *Klf2*, *Esrrb*, *Dppa3*, *Tcfcp2l1*, and *Prdm14*). Student's *t*-test was applied to examine the significance of the expression difference between two distinct XCI groups. (*E*) Overview of the activity of Chr Xs and expression of pluripotency genes for *Sox2*, *Esrrb*, and *Prdm14* in each cell.

To explore the genes underlying XCI states, we first compared the expression difference between uninitiated and initiated (ongoing and completed) XCI groups for each condition. We identified 69 (including the TFs *Hmgb3* and *Zic3*), 37 (including the TF *Pias2*), and 17 (including the TF *Zfp157*) DEGs (adjusted *P* < 0.01) in conditions of ES2i.F, ES.F, and delayed.Epi.F, respectively. Epi.F and Neuron.F conditions were not considered, because Epi.F only had completed-XCI cells and Neuron.F contained only four uninitiated-XCI cells. Nonetheless, most of those DEGs were specific to the corresponding conditions (Fig. 4A), suggesting that influence of XCI on gene expression was associated with the developmental states. Notably, the TFs *Hmgb3*, *Zic3*, and *Pias2* were significantly down-regulated in initiated (ongoing and completed) XCI cells in ES2i and ES states compared to uninitiated-XCI cells, and their reduced expression contributed to the differentiation of stem cells (Nemeth et al. 2006; Lim et al. 2007; Hong et al. 2009).

To further examine the association between XCI and differentiation, we overlaid the activity of Chr Xs onto each cell in the previous PCA. Two delayed.Epi.F cells with completed-XCI progressed into the cluster of Epi.M and Epi.F cells (arrows in Fig. 4B). Surprisingly, some delayed.Epi.F cells with completed-XCI (red dots) remained in the ES cluster. However, Epi.F cells had completed XCI and clustered together with Epi.M cells, indicating that there might be a time-lag between XCI and differentiation progression in single cells. Likewise, four Neuron.F cells with uninitiated-XCI were close to the ES cell cluster but further away from other Neuron cells (arrows in Fig. 4B), thus representing partly undifferentiated cells mixed in the neuronal population.

Remarkably, all aforementioned differentiation markers were negatively correlated with the activity of Chr Xs (*P* < 0.05) (Supplemental Fig. S5), and all pluripotency factors were positively correlated with the activity of Chr Xs in all female cells (*P* < 0.05) (Fig. 4C). This indicated that the expression of differentiation markers was gradually up-regulated, whereas pluripotency factors were generally down-regulated with XCI progression. Furthermore, cells within uninitiated-XCI groups expressed pluripotency factors at significantly higher levels compared to cells within initiated (ongoing and completed) XCI groups (Student's *t*-test, *P* < 0.01) (Fig. 4D). However, there was no significant difference in expression between uninitiated- and initiated-XCI groups in delayed.Epi.F condition. Nonetheless, expression of pluripotency markers for both uninitiated- and initiated-XCI groups of delayed.Epi.F were significantly higher than that of Epi.F completed-XCI group (Fig. 4D). Some female cells that had completed XCI in ES.F, delayed.Epi.F, and Neuron.F conditions still expressed pluripotency factors at relatively high levels (red ones with activity of Chr Xs around 1, e.g., *Sox2*, *Esrrb*, and *Prdm14*) (Fig. 4E) and Supplemental Fig. S6 for *Nanog*, *Pou5f1*, *Dppa3, Klf2*, and *Tcfcp2l1*. This phenomenon was predominantly found in delayed.Epi.F cells compared to Epi.F cells and suggests that additional time is needed to further down-regulate the expression of pluripotency factors after the completion of XCI for those cells. Consequently, our findings demonstrate the asynchronicity between loss of pluripotency and XCI progression at the single-cell level, although these processes are globally correlated.

### Transcriptional signature underlying the delayed progression of Epi female cells

**Figure 5.**
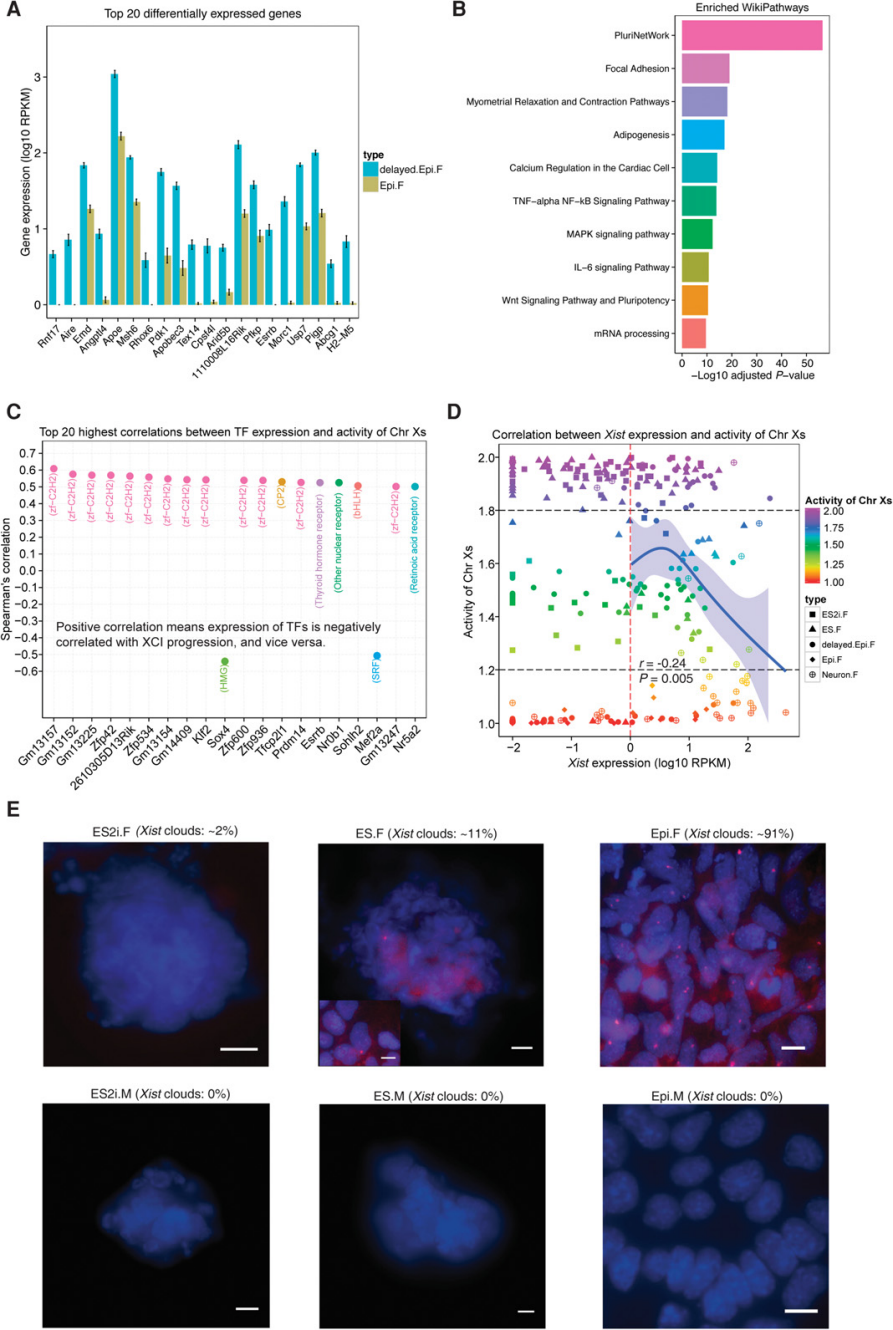
Underlying factors associated with delayed progression of mEpiSCs and heterogeneity in expression of *Xist*. (*A*) Top 20 DEGs between delayed.Epi.F and Epi.F cells (sorted by adjusted *P*, mean ± SEM). Cutoff: adjusted *P* < 0.01. (*B*) Top 10 significantly enriched WikiPathways for the DEGs between delayed.Epi.F and Epi.F cells. (*C*) Top 20 highest correlations between TF expression and the activity of Chr Xs. The family of each TF is shown in the parentheses. Spearman's correlations were with adjusted *P* < 0.01. (*D*) Spearman's correlation between *Xist* expression and the activity of Chr Xs. Confidence interval of 95% for the curve of Natural Spline is shown. (*E*) RNA FISH of *Xist* shown for female and male ES2i, ES, and Epi cells. *Xist* clouds are in red, and nuclei are in blue (DAPI staining). For ES.F cells, *Xist* clouds are shown in two different morphologies (colony and the metastable cells). The percentage of *Xist* clouds was calculated per 100 cells from multiple images (*n* = 6) per condition. The *Xist* clouds were imaged with 0.3 μm *z*-stack. (Scale bar) 10 μm.

We further investigated the underlying factors accounting for the delayed progression of Epi female cells. A total of 1528 up-regulated genes (96 TFs) and 1853 down-regulated genes (140 TFs) were identified when comparing Epi.F to delayed.Epi.F cells (adjusted *P* < 0.01). Strikingly, the top 20 DEGs including TFs *Aire*, *Rhox6*, *Arid5b*, and *Esrrb* were all significantly down-regulated in Epi.F cells (Fig. 5A). Moreover, the pluripotency factors *Sox2*, *Nanog*, *Klf2*, *Dppa3*, *Tcfcp2l1*, and *Prdm14* were also significantly down-regulated in Epi.F cells, whereas the differentiation markers *Fgf5*, *Eomes*, and *Cer1* were significantly up-regulated (Supplemental Fig. S7). These DEGs were enriched in the WikiPathways (Kutmon et al. 2016) such as PluriNetWork, Focal Adhesion, MAPK signaling pathway, and Wnt signaling pathway (adjusted *P* < 0.01) (Fig. 5B). MAPK and Wnt signaling pathways have been shown to be crucial for mESCs to exit the pluripotency state (Schulz et al. 2014). We have now identified pathways that may be responsible for the delayed progression of female mEpiSCs.

### Transcription factors that are highly correlated with XCI progression

TFs play important roles in regulating gene expression. We found that most of the top 20 TFs displaying the highest correlation with the activity of Chr Xs across female cells in all conditions were from the zf−C2H2 family (zinc finger, C2H2 type; Spearman's correlation |*r*| > 0.5 and adjusted *P* < 0.01) (Fig. 5C). Strikingly, pluripotency factors *Zfp42* (also known as *Rex1*), *Klf2*, *Tfcp2l1*, *Prdm14*, and *Esrrb* were among the top 20 TFs and highly positively correlated with the activity of Chr Xs (*r* > 0.5 and adjusted *P* < 0.01) (Fig. 5C). In contrast, *Sox4* and *Mef2a* were negatively correlated with the activity of Chr Xs (Fig. 5C). Therefore, these TFs could be involved in regulating their target genes to influence XCI progression.

### Heterogeneity in expression of *Xist* and *Tsix*

Because *Xist* is believed to play a central role in regulating XCI, we further analyzed the correlation between *Xist* expression and XCI progression at single-cell resolution. Only the female cells with *Xist* expression ≥1 RPKM were used to mitigate technical noise. We did not observe a high correlation between *Xist* expression and the activity of Chr Xs across female cells (*r* = −0.24, *P* = 0.005) (Fig. 5D). Most female cells with initiated-XCI expressed *Xist*. RNA FISH detected *Xist* clouds only in a small subset of ES2i.F (∼2%) and ES.F (∼11%) cells but in the majority of Epi.F (∼91%) cells (Fig. 5E), which further corroborated our allelic expression analyses. No *Xist* clouds were observed in corresponding male cells (Fig. 5E).

*Tsix* as the antisense transcript of *Xist* is transcribed to prevent *Xist* from silencing the active X Chromosome. We found that *Tsix* expression was positively correlated with *Xist* across different cell states except for ES2i.F cells (*r* ≥ 0.5, *P* < 0.05) (Supplemental Fig. S8A). This might be because most ES2i.F cells did not initiate XCI. In contrast, we did not observe significant expression correlations between *Xist* and *Tsix* in male cells (Supplemental Fig. S8B). Furthermore, female cells showed significantly higher expression levels of *Xist* and *Tsix* compared to that of the corresponding male cells, except for in the ES2i condition (*P* < 0.05) (Supplemental Fig. S8C,D). Spearman's correlation between *Tsix* expression and the activity of Chr Xs was also not high (*r* = −0.2, *P* = 0.003) (Supplemental Fig. S8E). Thus, both *Xist* and *Tsix* expression were heterogeneous in different XCI states at the single-cell level.

### Allelic expression of pluripotency genes

**Figure 6.**
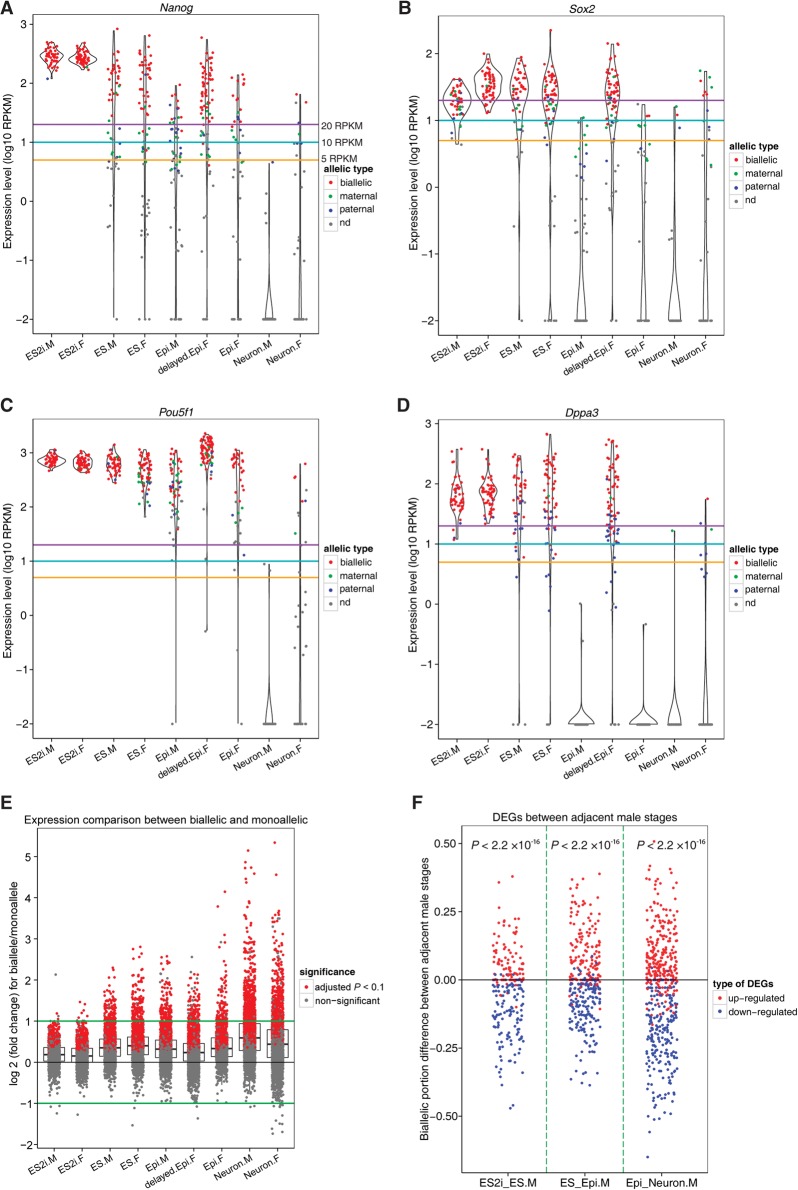
Allelic expression of pluripotency genes and contribution of allelic composition to differential expression. (*A*–*D*) Allelic expression profile for pluripotency genes *Nanog*, *Sox2, Pou5f1*, and *Dppa3*. Gold, cyan, and purple lines indicate the expression levels of 5, 10, and 20 RPKM. (*E*) Expression level comparison for each gene between biallelic and monoallelic fashions. Only those autosomal genes that are expressed at 20 or more RPKM in cell types with five or more bialleles and five or more monoalleles were considered. Student's *t*-test was applied to examine whether expression level of genes in a biallelic way was significantly higher than that in a monoallelic way. (*F*) Relationship between biallelic expression and up-/down-regulated genes between two adjacent male stages. *X*-axis denotes the comparing groups: ES2i versus ES, ES versus Epi, and Epi versus Neuron. *Y*-axis represents the biallelic fraction difference of cells between two comparing groups. Exact binomial test was applied to check the significance. Only those autosomal genes with expression level of 20 or more RPKM in at least 60% of a given cell type were considered. (M) male; (F) female; (nd) not detected.

The allelic expression of *Nanog* is still controversial (Miyanari and Torres-Padilla 2012; Faddah et al. 2013; Filipczyk et al. 2013). We found that allelic expression of *Nanog* as well as *Sox2*, *Pou5f1*, and *Dppa3* in cells with expression levels of 20 or more RPKM (purple line) was dominated by biallelic expression in all conditions (Fig. 6A–D; Supplemental Fig. S9, for *Esrrb* and *Prdm14*). Consistent with a reduced expression level of these genes, we observed a decrease in biallelic and an increase in monoallelic output across different conditions. Therefore, pluripotency genes are often expressed from both alleles in conditions in which their expression levels are high, whereas monoallelic expression is more prevalent once expression levels decrease.

### Genes show higher expression level with two alleles in use

We also found that a prominent portion of autosomal genes expressed in a biallelic fashion displayed significantly higher or comparable levels to monoallelic expression (adjusted *P* < 0.1, Student's *t*-test) (Fig. 6E; Supplemental Fig. S10A, for 20 and 10 RPKM criteria). This result further explained the aforementioned observation that highly expressed pluripotency genes are mainly biallelically expressed. Moreover, autosomal genes up-regulated in one condition generally had a higher proportion of cells expressed in a biallelic fashion and a lower proportion of cells expressed in a monoallelic fashion, and vice versa (*P* < 0.05) (Fig. 6F; Supplemental Fig. S10B–F, for 20 and 10 RPKM thresholds). Overall, up- and down-regulated genes could be reflected by their allelic expression status.

## Discussion

Studying the processes of pluripotency, differentiation, and XCI is crucial for understanding early development of mammals. To the best of our knowledge, this study is the first to systematically dissect the dynamics of XCI and the relationship with pluripotency and differentiation along developmental progression at the single-cell level with allelic resolution. A limitation of this study is that only one male and one female line were examined. However, we confirmed that there was negligible clonal effect between different lines (Supplemental Fig. S11). More ESC lines will be required to assess the impact of intercell variability and to study the dynamics of XCI associated with pluripotency and differentiation. A previous study suggested that mESCs grown in 2i and LIF closely resembled preimplantation epiblast E4.5 cells, but not E3.5 ICM based on 10–20 picked cells profiled by 96-gene arrays (Boroviak et al. 2014). However, we found that E3.5 ICM and E4.5 epiblast cells were similar in transcriptomic profile, and both were clustered nearby ES2i cells rather than to other cells.

Moreover, to date, no previous studies have reported genes specially enriched in ES cells compared simultaneously to ES2i, Epi, and Neuron cells. Here, we identified 130 genes enriched in ES cells, including TFs *Aire*, *Rarg*, *Wt1*, *Prdm5*, and *Tcfl5*. *Aire* is a well-known immune response gene that also promotes self-renewal through regulation of microRNA (Gu et al. 2010; Bin et al. 2012). *Rarg* is a retinoic acid receptor that is involved in reprogramming of somatic cells to induced pluripotent stem cells through retinoid acid signaling (Wang et al. 2011).

It is widely accepted that two active X Chromosomes are one of the key features of naïve female ESCs, while primed ESCs and differentiated ESCs only maintain one active X Chromosome. Although we did not observe that any ES2i.F cells had completed XCI, ∼30% of these had ongoing XCI. ES.F cells grown in the conventional serum/LIF condition were more heterogeneous in their XCI states, with ∼6% displaying completed-XCI. We captured two stages of EpiSCs—delayed.Epi.F cells with more heterogeneous XCI states, and extendedly cultured Epi.F cells with completed-XCI. Differentiation of ESCs to neurons is considered an asynchronized process. This asynchronicity was also reflected in their XCI states, with a few less differentiated ESCs that still had two active X Chromosomes. We believe this is the first extensive study on the heterogeneity of XCI states at the single-cell level during the differentiation process.

Schulz et al. suggested that two active X Chromosomes in female ESCs could block exit from the pluripotent state by modulating of MAPK and GSK3/Wnt pathways. We further confirmed that the DEGs between delayed.Epi.F and Epi.F cells were enriched in several pathways including MAPK and Wnt signaling pathways. Our newly identified PluriNetWork, Focal Adhesion, and TNF–alpha NF–kB signaling pathways may also regulate the exiting of pluripotency in female mESCs. It has been shown that disruption of epigenomic status in cultured mESCs impacts the expression of genes associated with development (Marks et al. 2012), and hypomethylation may exert a selection pressure for abnormal cells with deletion in one or two X Chromosomes (Zvetkova et al. 2005). Therefore, it is important to use early passages of female mESCs and correlate their developmental states with the corresponding male cells as well as with in vivo embryonic cells to ensure normal developmental progression. The delayed progression of female Epi cells compared to male Epi cells could also result from a lack of certain growth factors in the cultures. However, we cannot exclude that such a delay may exist shortly during peri-implantation (i.e., E4.5–E5) in female embryos.

We used allelic expression of maternal and paternal X Chromosomes to define the most accurate XCI states so far without only relying on *Xist* expression. We confirmed that XCI took place over the entire X Chromosome, which showed a gradual reduction of expression on the inactive X Chromosome during XCI progression. Overall, we observed that XCI progression was negatively correlated with pluripotency and positively correlated with differentiation. Strikingly, a fraction of cells with completed-XCI in ES.F, delayed.Epi.F, and Neuron.F conditions still expressed pluripotency factors, including *Sox2* and *Esrrb*. Thus, we provide the first evidence that XCI progression and down-regulation of pluripotency are not tightly synchronized at the single-cell level.

Notably, *Xist* expression is highly heterogeneous at the single-cell level among different XCI states. However, *Xist* showed an overall higher expression in cells with initiated-XCI compared to those with uninitiated-XCI. We observed some cells with initiated-XCI but low *Xist* expression, suggesting that these could undergo XCI with low *Xist* expression (Kalantry et al. 2009). However, *Xist* expression might possibly be underestimated in these cells because it is difficult to release all the *Xist* transcripts coated on the inactive X Chromosome for sequencing. We separately detected 2%, 11%, and 91% *Xist* clouds in ES2i.F, ES.F, and Epi.F cells. This is in agreement with our computational analysis that ∼6% ES.F and almost 100% Epi.F cells finished XCI. RNA FISH may not be sensitive enough to detect the female cells that have just initiated but not finished XCI; thus, only a very small portion of ES2i.F cells showed *Xist* clouds.

Allelic expression of pluripotency genes has never been systematically analyzed at the single-cell level. Our extensive single-cell allelic analysis revealed that key pluripotency genes were highly expressed and mainly displayed biallelic expression in the ground state condition of ES2i. In contrast, pluripotency genes in the conventional ES condition displayed lower expression and exhibited increased monoallelic expression. Moreover, autosomal genes generally displayed higher or comparable levels in biallelic than in monoallelic expression. These observations further extend our previous observation that the likelihood of observing monoallelic or biallelic expression of a gene in a cell depends on the gene expression level (Deng et al. 2014; Reinius and Sandberg 2015).

In summary, our single-cell analyses with allelic resolution provide novel insights into the dynamics of XCI progression and its asynchronized relationship with differentiation and pluripotency during developmental progression. We also revealed novel genes and pathways that are involved in these processes.

## Methods

### Derivation and culture of mESCs

The two mESC lines (one male, one female, passage 3) used in this study were derived from E4 blastocysts of crossbred embryos (female C57BL/6J × male CAST/EiJ). The derivation and culturing were performed as previously described (Meissner et al. 2009). For ES2i culture, mESCs were adapted to 2i culture conditions by growing them in gelatin-coated flasks in N2B27 medium (50% neurobasal medium [Gibco], 50% DMEM/F12 [Gibco], 2 mM L-glutamine [Gibco], 0.1 mM β- mercaptoethanol, NDiff Neuro2 supplement [Millipore], B27 serum-free supplement [Gibco]) supplemented with 1000 units/mL LIF and 2i (3 µM Gsk3 inhibitor CT-99021, 1 µM MEK inhibitor PD0325901) for two to six passages (passaged with accutase, Gibco). To induce differentiation toward EpiSCs, serum/LIF ESCs were plated in N2B27 medium supplemented with 8 ng/mL Fgf2 (R&D) and 20 ng/mL Activin A (R&D) on FBS coated (overnight) tissue culture plates at a cell density of 1 × 10^4^ cells/cm^2^. The cells grew to confluence; then after a pronounced crisis, only colonies containing flat EpiSC morphology survived and could be passaged using Collagenase IV (Thermo Scientific). Colonies can be passaged every 4 or 5 d at a 1:4–1:6 split.

### Extended culture of female delayed EpiSCs

Both male and female EpiSC conversions were performed using the same scheme of maintenance and passaging. Conversions were originally carried out for four passages in EpiSC conditions before being manually picked for sequencing. Although the converted male EpiSCs exhibited a post-implantation profile, the female cells reflected more diffuse delayed intermediate cells. The delayed female EpiSCs were cultured an additional three passages before being manually picked for sequencing, and exhibited a transcriptional profile indicative of primed post-implantation pluripotency.

### Differentiation of mESCs into post-mitotic neurons

mESCs were differentiated to neurons (primarily motor neurons and interneurons) via embryoid body formation followed by monolayer culture with growth factors. The protocol has been previously described in detail (Allodi and Hedlund 2014; Nichterwitz et al. 2016).

### Collection of in vivo embryonic cells

Preimplantation mouse E3.5 and E4.5 embryos were derived from crossing between male CAST/EiJ and female C57BL/6J. Isolation of single cells was performed as described in Deng et al. (2014). Post-implantation mouse E5.5 embryos were derived from pure C57BL/6J breeding. E5.5 epiblast cells were isolated according to the protocol described previously (Chenoweth and Tesar 2010).

### Preparation of single-cell sequencing libraries

Libraries for RNA-seq were prepared by using Smart-seq2 combined with Tn5 tagmentation (Picelli et al. 2013, 2014). Single-end 43-bp reads were generated using HiSeq 2000 (Illumina).

### Read mapping and gene expression quantification

Sequencing reads were mapped to the mouse reference genome (mm10) using STAR (Dobin et al. 2013) (version 2.4.1) with parameter --outSAMstrandField intronMotif. The number of unique reads for each gene of Ensembl 78 was calculated by featureCounts (Liao et al. 2014) (version 1.4.6) with default parameters. We further quantified and normalized gene expression across different samples using Cufflinks and Cuffnorm (Trapnell et al. 2010) (version 2.2.1) with parameters -library-norm-method geometric, -GTF, -u and -b enabled. To do the PCA clustering based on variable genes, the functions of varianceStabilizingTransformation and plotPCA in DESeq2 (Love et al. 2014) (version 1.6.3) were used. To guarantee the authenticity of E3.5 ICM cells as well as E4.5 and E5.5 epiblast cells, we only used those cells with expression of core pluripotency markers of *Nanog*, *Sox2*, and *Pou5f1* greater than 1 RPKM. We obtained the mouse TFs from AnimalTFDB 2.0 database (Zhang et al. 2015). More details can be found in Supplemental Methods.

### Differential gene expression calling and KEGG pathway analysis

To compare the gene expression between male and female cells, we conducted differential expression analysis using SCDE (Kharchenko et al. 2014) (version 1.2.1). The male and female cells of each condition were compared. We excluded seven undifferentiated neuron female cells in differential expression according to the following allelic expression analysis. To obtain the gene expression changes across different developmental states, we divided those conditions into three groups (ES2i versus ES, ES versus Epi, and Epi versus neuron). Differentially expressed genes (DEGs) were defined as adjusted *P* < 0.01. After the determination of DEGs, we conducted the KEGG pathway analysis using WebGestalt (Wang et al. 2013). Only those pathways with an adjusted *P* < 0.01 were considered statistically significant.

### Allelic expression calling

Allelic expression calling was carried out using our previous pipeline (Deng et al. 2014). SNPs of C57BL/6J and CAST/EiJ that showed consistent and reliable allelic expression in our previous study (Deng et al. 2014) were considered. We further calculated the ratio of maternal X Chromosome expression in each cell based on the number of uniquely mapped allele informative reads: C57BL/(C57BL + CAST). We retrieved the XCI state of each cell by converting the maternal expression ratio: (1) if the maternal expression ratio was 0.5 or greater, (activity of Chr Xs) = 1/(maternal expression ratio); and (2) if the maternal expression was less than 0.5, (activity of Chr Xs) = 1/1−(maternal expression ratio). Thus, the values for the activity of Chr Xs in female cells ranged from 1 (one active Chr X) to 2 (two active Chr Xs). More details can be found in Supplemental Methods.

### Calculating the XCI progression across the X Chromosome

To examine XCI progression in female cells, we compared gene expression (measured by the uniquely mapped SNP-containing counts) of the inactive X Chromosome with that of the active X Chromosome using a moving window size of 10 genes on average. The ratio of uniquely mapped SNP-containing reads of the inactive X Chromosome divided by that of the active X Chromosome for each gene was calculated. Subsequently, we used an average of 10 genes as a moving window to plot the ratio as a line across the entire X Chromosome. More details can be found in Supplemental Methods.

### RNA FISH

Cells were cultured as described above and then seeded on an eight-well chamber slide. RNA FISH was conducted and imaged as previously described (Petropoulos et al. 2016). A mouse FISH probe XIST Quasar 570 (125 nM; SMF-3011-1; BioSearch Technologies) was used.

## Data access

The sequencing data from this study have been submitted to the NCBI Gene Expression Omnibus (GEO; http://www.ncbi.nlm.nih.gov/geo/) under accession number GSE74155.

## Supplementary Material

Supplemental Material
