# Supplementary material for: Single-cell analyses of X Chromosome inactivation dynamics and pluripotency during differentiation
Source: Genome Res. 2016 Oct;26(10):1342–54. doi: 10.1101/gr.201954.115 (PMC5052059; doi:10.1101/gr.201954.115)
Supplement: Supplemental Material [file supp_26_10_1342__index.html]

Single-cell analyses of X Chromosome inactivation dynamics and pluripotency during differentiation — Supplemental Material 

# Single-cell analyses of X Chromosome inactivation dynamics and pluripotency during differentiation

## Supplemental Material

- Supplemental\_Table\_S1.xlsx
- Supplemental\_Table\_S2.xlsx
- Supplemental\_Table\_S3.xlsx
- Supplemental\_Table\_S4.xlsx
- Supplemental\_Table\_S5.xlsx
- Supplemental\_Files\_20160729.docx
