# Supplementary material for: Single-cell analyses of X Chromosome inactivation dynamics and pluripotency during differentiation
Source: Genome Res. 2016 Oct;26(10):1342–54. doi: 10.1101/gr.201954.115 (PMC5052059; doi:10.1101/gr.201954.115)
Supplement: Supplemental Material [file supp_gr.201954.115_Supplemental_Files_20160729.docx]

Supporting Online Material for:

**Single-cell analyses of X Chromosome inactivation dynamics and pluripotency during differentiation**

Geng Chen^1,2^, John Paul Schell^3^, Julio Aguila Benitez^1,4,6^, Sophie Petropoulos^1,3,6^, Marlene Yilmaz^1^, Björn Reinius^1^, Zhanna Alekseenko^1^, Leming Shi^2^, Eva Hedlund^4^, Fredrik Lanner^3^, Rickard Sandberg^1,5^, Qiaolin Deng^1*^

***To whom correspondence should be addressed. Email:** [**Qiaolin.Deng@ki.se**](mailto:Qiaolin.Deng@ki.se)

**This PDF file includes: Supplemental Methods, and Supplemental Figures 1-11**

**Supplemental Methods**

**Read mapping and gene expression quantification**

RNA-seq reads of each cell were mapped to the mouse reference genome (mm10) using STAR (Dobin et al. 2013) (version 2.4.1) with parameter --outSAMstrandField intronMotif. We removed cells with low number of sequencing reads (< 800,000) and a low mapping ratio (< 60%). We also confirmed the karyotypes of all cells based on the allelic expression profile (see below) of maternal and paternal chromosomes and only cells with correct karyotypes were included in the analysis. The number of uniquely mapped reads for each gene of Ensembl 78 was calculated by employing featureCounts (Liao et al. 2014) (version 1.4.6) with default parameters. We further quantified and normalized gene expression across different samples using Cufflinks and Cuffnorm (Trapnell et al. 2010) (version 2.2.1) with parameters of -library-norm-method geometric, -GTF, -u and –b enabled. For Principle Component Analysis (PCA) clustering, we first applied the function of varianceStabilizingTransformation in DESeq2 (Love et al. 2014) (version 1.6.3) to the matrix that contains uniquely mapped read counts of each gene feature for all cells. Next, we did the PCA clustering using the function of plotPCA in DESeq2, which was based on the top 500 variable genes in expression by default. To validate the robustness of the PCA clustering, we also separately tried the PCA based on the top 200 and 1,000 variable genes, and obtained similar clustering results. To guarantee the authenticity of E3.5 ICM cells as well as E4.5 and E5.5 epiblast cells, we only used those cells with expression of core pluripotency markers of *Nanog*, *Sox2* and *Pou5f1* greater than 1 RPKM. We obtained the mouse transcription factors (TFs) from AnimalTFDB 2.0 database (Zhang et al. 2015).

**Expression quantification of *Xist* and *Tsix***

We are aware that *Xist* and *Tsix* have overlapping sequences due to the antisense nature of *Xist* and *Tsix* and are confident that the expression quantification for *Xist* and *Tsix* is accurate. In order to increase the accuracy of gene expression quantification, we supplied the mouse Ensembl reference transcript annotation (version 78, GTF format) and enabled the parameters of –GTF (to use GTF annotation to guide assembly), -u (to more accurately weight reads mapping to multiple locations in the genome) and –b (for read mapping bias detection and correction), which allowed Cufflinks to use the reference annotation based transcript (RABT) assembly algorithm (Roberts et al. 2011). These parameters together accurately estimated the gene expression since alignments that are structurally compatible with the reference transcript annotation were only used. Furthermore, if the antisense nature of *Xist* and *Tsix* would influence the expression quantification in our study, we should also have observed a significant correlation between *Xist* and *Tsix* in male cells as in female cells, but this was not found (Supplemental Fig. S8A,B).

**Allelic expression calling**

To explore the allelic expression profile of those cultured embryonic cells, we first carried out allelic expression calling using our previous pipeline (Deng et al. 2014). Only those confirmed SNPs of C57BL/6J (mother) and CAST/EiJ (father) in our previous study (Deng et al. 2014) were considered in this study. Those SNPs were from the whole genome sequencing of mouse strain (Keane et al. 2011), but only those ones that can be validated in the RNA-seq data from pure C57BL/6J and CAST/EiJ cells were used (Deng et al. 2014). In total, 828,221 SNPs located in transcribed regions that showed consistent and reliable allelic expression demonstrated in our previous study were used in this work.

We further calculated the ratio of maternal expression for X Chromosome in each cell based on the uniquely mapped allele informative reads: C57BL/(C57BL + CAST), the number of uniquely mapped reads that contain C57BL SNPs divided by the number of uniquely mapped reads that harbor C57BL SNPs and add that of CAST. In order to determine the XCI state in each cell, we converted the maternal expression ratio as bellow: i) if the maternal expression ratio ≥ 0.5, (activity of Chr Xs) = 1/(maternal expression ratio); ii) if the maternal expression < 0.5, (activity of Chr Xs) = 1/1-(maternal expression ratio). The values for activity of Chr Xs in female cells ranged from 1 (one active X Chromosome) to 2 (two active X Chromosomes).

**Calculating the XCI progression across X Chromosome**

To examine the XCI progression in female cells, we compared the gene expression (measured by the uniquely mapped SNP-containing read counts) of inactive X Chromosome with that of active X Chromosome using a moving window size of ten genes on average. The inactive and active X Chromosomes were characterized according to the maternal expression ratio (as described above) of each cell. A maternal expression ratio for a female cell < 0.5 indicated that this cell was inactivating the maternal Chr X (C57BL), and vice versa. First, the female cells were divided into three distinct XCI state groups: i) cells with uninitiated-XCI (1.8 < activity of Chr Xs ≤ 2); ii) cells with ongoing-XCI (1.2 < activity of Chr Xs ≤ 1.8); and iii) cells with completed-XCI (1 ≤ activity of Chr Xs ≤ 1.2). The criterion was based on our observation that expression ratio of maternal/paternal autosomes has up to 5% fluctuation. Considering this, we allowed ~5% fluctuation for expression ratios of both maternal and paternal X Chromosomes. As cells with uninitiated-XCI have two active X Chromosomes and their maternal expression ratios were around 0.5, thus we defined uninitiated-XCI cells as 1.8 < activity of Chr Xs ≤ 2 (conversion: 1/(0.5+0.05) ≈ 1.8 or 1/(1-(0.5-0.05)) ≈ 1.8). For cells with completed-XCI, there just remain one active X Chromosome and their maternal expression ratios were around 0 (inactive maternal X Chromosome) or 1 (inactive paternal X Chromosome). We allowed a 15% fluctuation for the expression ratio of inactive X Chromosome as some escape genes still have certain expression levels from the inactive X Chromosome, which resulted in completed-XCI cells have 1 ≤ activity of Chr Xs ≤ 1.2 (conversion: 1/(1-0.15) ≈ 1.2 or 1/(1-(0+0.15)) ≈ 1.2). Then we defined those cells with 1.2 < activity of Chr Xs ≤ 1.8 as ongoing-XCI. We further confirmed our classifications for these three distinct XCI groups were reasonable based on the XCI progression analysis across the entire X Chromosome. To minimize the bias of cell-to-cell variability, we only used those genes with the median value >= 2 uniquely matched SNP-informative reads on active X Chromosome in each XCI state group for each condition. The ratio of uniquely mapped SNP-containing reads of inactive X Chromosome divided by that of active X Chromosome for each gene was calculated. An average of ten genes as a moving window was utilized to plot the ratio (depicted as line) across the whole X Chromosome for each XCI state group in corresponding figures.

**Supplemental Figures**

**Supplemental Fig. S1.** PCA clustering based on the top 200 and 1,000 variable genes. (A and B) are PCAs for all the cultured cells of distinct developmental states based on the top 200 and 1,000 variable genes, respectively. (C and D) are PCAs for both cultured (*in vitro*) and *in vivo* cells of distinct developmental states based on the top 200 and 1,000 variable genes, respectively.

**Supplemental Fig. S2.** KEGG pathways for genes with enriched expression in each developmental state. (A-D) Top significantly enriched KEGG pathways for genes highly enriched in ES2i, ES, Epi and Neuron conditions in expression, respectively. Adjusted *P* < 0.01.

**Supplemental Fig. S3.** Enriched biological processes of GO terms for differentially expressed genes. (A-D) Top ten biological processes of GO terms for differentially expressed genes between male and female cells of ES2i, ES, Epi and Neuron, respectively.

**Supplemental Fig. S4.** Allelic expression profile for known genes that escape XCI. (A, B, C and D) are the expression levels of *Kdm5c*, *Kdm6a*, *Ddx3x* and *2610029G23Rik* measured by the SNP-containing counts from the inactive Chr X in different XCI state groups. Female cells were divided into three distinct XCI state groups: (i) uninitiated-XCI (1.8 < activity of Chr Xs ≤ 2); (ii) ongoing-XCI (1.2 < activity of Chr Xs ≤ 1.8); and (iii) completed-XCI (1 ≤ activity of Chr Xs ≤ 1.2). ES2i.F condition only contained the cells with uninitiated- and ongoing-XCI, while Epi.F condition just had the cells with completed-XCI.

**Supplemental Fig. S5.** Spearman’s correlation between expression of differentiation markers and the activity of Chr Xs. All these differentiation makers were significantly negatively correlated with the activity of Chr Xs, indicating their expression levels were gradually increased with XCI progression.

**Supplemental Fig. S6.** Relationship between the activity of Chr Xs and expression level of pluripotency markers of *Nanog*, *Pou5f1*, *Dppa3*, *Klf2* and *Tcfcp2l1* at the single-cell level.

**Supplemental Fig. S7.** Differentially expressed pluripotency genes and differentiation markers between delayed.Epi.F and Epi.F cells. Adjusted *P* < 0.01.


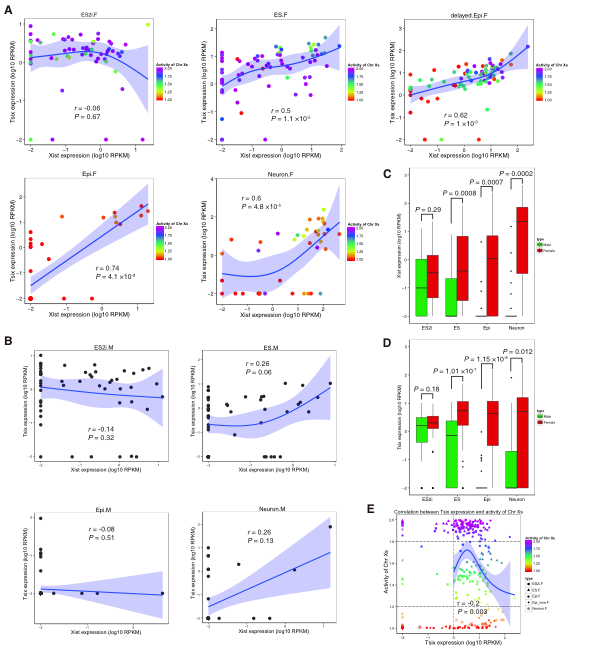


**Supplemental Fig. S8.** Expression profile of *Xist* and *Tsix* in male and female cells. (A) Spearman’s correlation between the expression of *Xist* and *Tsix* in female cells of each condition. (B) Spearman’s correlation between *Xist* and *Tsix* in male cells of each condition. (C) Expression comparison of *Xist* between male and female cells. (D) Expression comparison of *Tsix* between male and female cells. (E) Spearman’s correlation between *Tsix* expression and the activity of Chr Xs. 95% confidence interval for the curve of Natural Spline was shown in Figure S8 A, B and E.

**Supplemental Fig. S9.** Allelic expression profile for pluripotency genes *Esrrb* and *Prdm14*.

**Supplemental Fig. S10.** Gene expression characteristics of biallelic and monoallelic fashion. (A) Expression level comparison between biallelic and monoallelic of each gene. Only those autosomal genes expressed higher than 10 RPKM in each condition of cells with ≥ 5 bialleles and ≥ 5 monoalleles were considered. Student’s t-test was applied to check whether the expression of genes in a biallelic way was significantly higher than that of in a monoallelic way. Red dots represent significant genes (adjusted *P* < 0.1, FDR correction), whereas gray dots denote non-significant genes. (B) Relationship between biallelic portion difference of cells and up/down-regulated autosomal genes between male and female. Y-axis denotes the proportion of cells with biallelic expression in male subtracted by that of female. (C) Relationship between biallelic portion difference of cells and up/down-regulated autosomal genes between adjacent female stages. X-axis denotes the comparing groups: ES2i versus ES, ES versus Epi and Epi versus neuron. Y-axis represents the difference of proportion of cells with biallelic expression between two comparing groups. (B, C) are the results with cutoff ≥ 20 RPKM in at least 60% of a certain type of cells, while (D-F) are the results with cutoff ≥ 10 RPKM. Exact binomial test was applied to check the significance. Note: M and F are male and female cells, respectively.

**Supplemental Fig. S11.** PCA for the four different male lines in ES condition. The clustering was based on the top 500 variable genes. Cells of four different male lines were clustered together and no clonal effect was found among these lines. “clone_1” is the male line used in the study.

**References**

Deng Q, Ramsköld D, Reinius B, Sandberg R. 2014. Single-cell RNA-seq reveals dynamic, random monoallelic gene expression in mammalian cells. *Science* **343**: 193–196.

Dobin A, Davis CA, Schlesinger F, Drenkow J, Zaleski C, Jha S, Batut P, Chaisson M, Gingeras TR. 2013. STAR: ultrafast universal RNA-seq aligner. *Bioinformatics* **29**: 15–21.

Keane TM, Goodstadt L, Danecek P, White MA, Wong K, Yalcin B, Heger A, Agam A, Slater G, Goodson M, et al. 2011. Mouse genomic variation and its effect on phenotypes and gene regulation. *Nature* **477**: 289–294.

Liao Y, Smyth GK, Shi W. 2014. featureCounts: an efficient general purpose program for assigning sequence reads to genomic features. *Bioinformatics* **30**: 923–930.

Love MI, Huber W, Anders S. 2014. Moderated estimation of fold change and dispersion for RNA-seq data with DESeq2. *Genome Biol* **15**: 550.

Roberts A, Pimentel H, Trapnell C, Pachter L. 2011. Identification of novel transcripts in annotated genomes using RNA-Seq. *Bioinformatics* **27**: 2325-2329.

Trapnell C, Williams BA, Pertea G, Mortazavi A, Kwan G, van Baren MJ, Salzberg SL, Wold BJ, Pachter L. 2010. Transcript assembly and quantification by RNA-Seq reveals unannotated transcripts and isoform switching during cell differentiation. *Nat Biotechnol* **28**: 511–515.

Zhang H-M, Liu T, Liu C-J, Song S, Zhang X, Liu W, Jia H, Xue Y, Guo A-Y. 2015. AnimalTFDB 2.0: a resource for expression, prediction and functional study of animal transcription factors. *Nucleic Acids Res* **43**: D76–81.
